# CDKL5 Deficiency Disorder (CDD)—Rare Presentation in Male

**DOI:** 10.3390/children9121806

**Published:** 2022-11-24

**Authors:** Małgorzata Rodak, Mariola Jonderko, Patrycja Rozwadowska, Magdalena Machnikowska-Sokołowska, Justyna Paprocka

**Affiliations:** 1 Students’ Scientific Society, Department of Pediatric Neurology, Faculty of Medical Sciences in Katowice, Medical University of Silesia, 40-752 Katowice, Poland; 2 Department of Diagnostic Imaging, Radiology and Nuclear Medicine, Faculty of Medical Science in Katowice, Medical University of Silesia, 40-752 Katowice, Poland; 3 Department of Pediatric Neurology, Faculty of Medical Sciences in Katowice, Medical University of Silesia, 40-752 Katowice, Poland

**Keywords:** CDKL5 deficiency disorder, epileptic encephalopathy, refractory epilepsy, children

## Abstract

CDKL5 deficiency disorder (CDD) is a developmental encephalopathy caused by pathogenic variants in the X-linked cyclin-dependent kinase 5 (CDKL5) gene. This rare disorder occurs more frequently in females than in males. The incidence is estimated to be approximately 1: 40,000–60,000 live births. So far, 50 cases have been described in boys. The clinical course in males tends to be more severe and is often associated with death in the first or second decade of life. The authors present an unreported 2.5-year-old male patient with drug-resistant epilepsy who was diagnosed with a de novo mutation in the *CDKL5* gene. First seizures developed in the fifth week of life and have progressed steadily since then. The child’s psychomotor development was strongly delayed, and generalized hypotonia was noticed since birth. Brain MRI showed areas of incomplete myelination, posterior narrowing of the corpus callosum, a pineal cyst of up to 3 mm, and open islet lids. Intensive antiseizure medications (ASMs), a ketogenic diet, and steroid therapy were not successful. Short-term improvement was achieved with the implantation of a vagal nerve stimulator (VNS). Due to the progressive course of the disease, the boy requires frequent modification of ASMs.

## 1. Introduction

*CDKL5* deficiency disorder (CDD) is a rare and often profound neurodevelopmental encephalopathy caused by pathogenic alterations in the *CDKL5* gene and a deficiency of functional cyclin-dependent kinase-like 5 (*CDKL5*) [1]. The translation product protein belongs to the serine/threonine kinase family and is widely distributed in the human body, with highest expression in the brain (cortex, hippocampus, cerebellum, thalamus, and brainstem), testes, and thymus gland. Although its role has not been precisely defined, it is now known that *CDKL5* plays an important role in the formation and maturation of neurons and also in the development and function of synapses [2,3]. The mutation rate in *CDKL5* is estimated to be approximately 1 in 40,000–60,000 live births [4]. This genetic disorder causes early-onset seizures, developmental delay, and severe intellectual disability. Seizures usually begin within the first three months after birth and are difficult to control with medications [5]. In addition, this disorder is characterized by hypotonia and cortical visual disturbances. It may also be accompanied by gastrointestinal symptoms, such as constipation, reflux, and air swallowing, which give rise to feeding problems. Other features that can be observed include poor eye contact, hand stereotyping, scoliosis, absent or limited speech, dysmorphic facial features, and autonomic disorders [4,6]. The clinical pictures for *CDKL5* disorders are not uniform and may be due to the type and location of the mutation and to the influence of epigenetic and environmental factors. *CDKL5* pathogenic variants are diagnosed in 8–16% of girls with early-onset epilepsy [7]. *CDKL5* is located on the X chromosome (Xp22), and *CDKL5* deficiency disorder (CDD) is four times more common in women than in men, suggesting that it is mainly a fatal mutation in male fetal life [2]. CDD in men has a more severe disease course and is often fatal in the first or second decade of life [8]. Since *CDKL5* is located on the X chromosome, men with CDD do not have functional *CDKL5* in any of their cells, while women have a mosaic of cells: about 50% of cells will express wild-type *CDKL5,* and 50% of cells will not express functional *CDKL5* (due to random X inactivation during development) [1]. Changes in the *CDKL5* gene were first linked to the human disease in 2004 by observing cohorts of patients with Rett syndrome who had no changes in the MeCP2 (methyl CpG 2 binding protein) gene. However, CDD is not, nor is it even a variant of, Rett syndrome, but a unique, distinct, and complex disorder that is particularly rare in the male population [7].

## 2. Case Report

**Figure 1:**
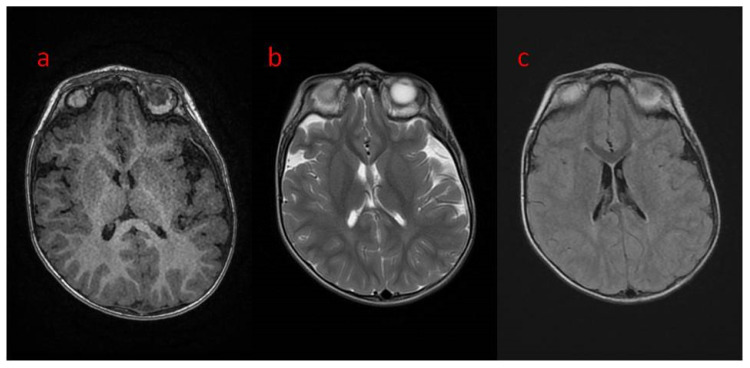
(**a**–**c**) Brain MRI in transverse planes: (**a**) normal in T1; (**b**,**c**) unfished myelinisation in peritrigonal white matter in (**b**) T2; (**c**) T2 FLAIR.

**Figure 2:**
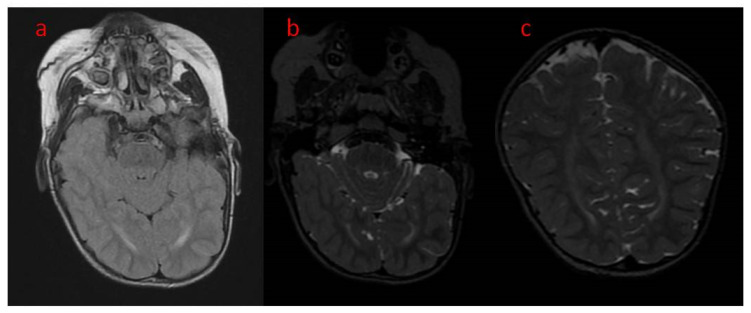
(**a**–**c**) Brain MRI, axial (**a**) T2 FLAIR (**b**) T2, coronal (T2) unfinished myelinisation of white matter surrounding occipital horns.

**Figure 3:**
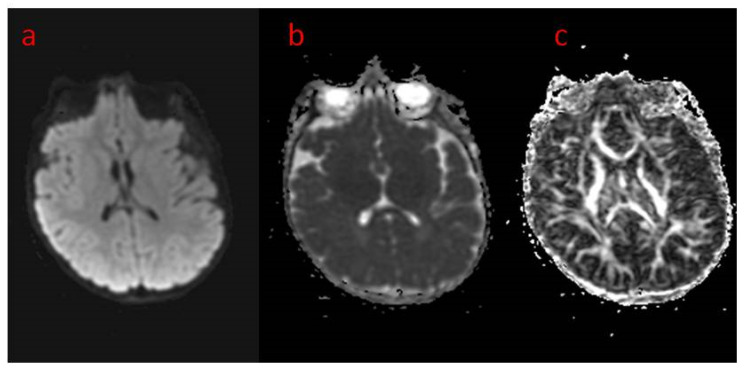
(**a**–**c**) Brain MRI in advanced sequences: (**a**) DWI diffusion weighted imaging; (**b**) ADC map—apparent diffusion coefficient map—areas of prolonged diffusion in peritrigonal white matter regions corresponding to unfinished myelinisation, (**c**) FA—fractional anisotropy sequence—symmetrical image of fibers of both hemispheres.

The presented case is a 2.5-year-old patient with epilepsy of genetic background. He was born from the first gestation, at 39 weeks by cesarean section because of breech position. The pregnancy was uneventful, with regular growth and fetal movements. His birth weight was 4630 g, with a body length of 56 cm and a head circumference of 39 cm. He scored 10 on the Apgar scale. The family history was negative except for the grandmother on the mother’s side, who had been diagnosed with schizophrenia. The patient’s psychomotor development was profoundly delayed, and he could not reach the milestones at age 2.5. Upon admission to hospital at age 2, the boy was in the cooing stage, he was not even raising his head or fixating on or following objects. In the neurological examination, the following were noted: plagiocephaly of the right parietal area, the cranial nerve functions that could be checked were intact, generalized hypotonia with preserved tendon reflexes (upper limbs > lower limbs), positive bilateral Babinski sign, and Chaddock’s and Oppenheim’s signs were slightly positive. He reacted poorly to environmental stimuli. He also clenched his fists and had a tendency to turn his head to the right. The patient has contractures in various joints, including the ankles, right knee, shoulder, left elbow, and to a slight degree in the hip joints. The child has been fed with blended, not solid meals. The first epileptic seizures were noticed at the age of 5 weeks as 30 s convulsions of the whole body accompanied by apnea. The seizures mainly occurred while falling asleep. After 2–3 months, the seizures began to occur with greater frequency and duration. With time, the boy experienced up to 30 seizures a day. They were dominated by epileptic spasms and myoclonus seizures, as well as focal motor seizures. The boy was repeatedly hospitalized for the intensification of his epileptic seizures. Brain CT at two months of age showed increased pericerebral spaces within the Sylvian fissure. In the fifth month of life, based on genetic work-up, a pathogenic variant of the *CDKL5* gene was confirmed, a de novo substitution of one nucleotide c.513C > G, resulting in a nonsense mutation with the formation of a premature STOP codon (p.Tyr171X) disrupting normal protein synthesis. The first EEG performed in the sixth month of life showed generalized paroxysmal discharges with a pattern resembling “burst-suppression” but without the typical episodes of bioelectrical silence, with normal sleep characteristics. A second EEG was performed in the boy’s second year of life. In the record of the brain’s bioelectric activity, there were continuous seizure changes described in the routine examination. During video EEG recording, limb tearing and incidents of eye rotation were observed. At that time, continuous paroxysmal discharges were present in the recording of bioelectrical activity of the brain. Brain MRI at age 2 showed areas of unfinished myelination in the lateral ventricular triangles and around the occipital horns (Figure 1a–c) and a small pineal cyst of up to 3 mm in size. Normal brain MR images in the standard sequences of the study are shown in Figure 2a–c. Figure 3 shows MR images in advanced sequence DWI, ADC map, and FA sequence. There was a preserved symmetrical image of the fibers of both hemispheres.

Extensive pharmacotherapy, including antiepileptic treatment (valproic acid, topiramate, vigabatrin, phenobarbital, levetiracetam, phenytoin, clobazam, clonazepam, nitrazepam, lamotrigine, carbamazepine, and zonisamide) and steroid therapy (adrenocorticotropic hormone, and methylprednisolone) were administered. However, due to the development of severe dyskinesias and dystonia, steroid therapy was discontinued. The patient repeatedly required modification of ASMs, as the drugs used did not bring significant clinical improvement. The patient is currently taking levetiracetam 2 mL-0-3.5 mL and lamotrigine, the dose of which, due to persistent seizures, has been increased from 0-0-1 mL to 1-0-1 mL. This modification initially yielded good results, but over time the symptoms began to worsen again.

Two months after the diagnosis of the *CDKL5* mutation, a vagal nerve stimulator (VNS) was implanted. The frequency of epileptic seizures was reduced by about half, but after about five months they became more frequent again, requiring modification of the neurostimulation parameters by the consulting neurosurgeon. Due to reluctance to drink and increased dystonic movements, the ketogenic diet was discontinued after 5 days. Since birth, the patient has been receiving rehabilitation; at present, he is without visual and verbal contact and not able to lift his head.

## 3. Discussion

CDKL5 disorder, originally classified as a variant of Rett syndrome, is now recognized as an independent disorder and classified as a developmental epileptic encephalopathy [9]. The diagnosis of CDKL5 disorders can be suspected based on history, symptoms, and physical examination, but only a molecular diagnosis will confirm the disorder as CDD, caused by loss-of-function variants in *CDKL5*. It should be suspected in patients with early-onset epilepsy, severe developmental delay, and poor response to antiepileptic medicaments [7]. CDKL5 disorders are confirmed using molecular genetic testing for *CDKL5* pathogenic variants or multigene panel testing for early-onset epilepsy [10,11]. Since some *CDKL5* variants are not pathogenic but benign, the variants must be considered pathogenic according to recognized pathogenicity assessment guidelines to confirm a diagnosis [11]. In most cases, pathogenic variants occur de novo. However, cases of parental mosaicism have been reported [7,9]. MRI brain imaging studies can reveal many nonspecific changes that may also be present in other diseases, such as brain atrophy, white matter hyperintensity, and ventriculomegaly. Brain changes are rarely present at the onset of the disease, and in some patients they do not appear at all. Therefore, neuroimaging studies may not be helpful for the diagnosis. The gender of the patient seems to matter, as brain imaging abnormalities have been found to be more common in males than in females and are associated with a more severe disease course [7,9,12].

CDKL5 deficiency disorder is a rare condition, more common in females than in males. To the best of our knowledge, 50 males with CDD have been described so far [13]. Affected boys tend to have a more severe phenotype than girls. The clinical picture in boys with CDD is mainly characterized by severe general developmental delay (SGDD), with impaired gross motor function, inability to acquire language, cortical visual impairment (CVI), and refractory epilepsy of early onset. It is worth mentioning that the clinical variability is probably genetically determined [9,12,13].

The presented patient had been diagnosed with a *CDKL5* pathogenic variant which has not yet been described in the literature. Table 1 presents 15 selected clinical cases of boys with the other *CDKL5* pathogenic variants and a new case. For comparison, we have chosen boys whose disease onset was around the first month of life. We are dealing with various pathogenic variants, most of which were created “de novo” (87.5%), as in the case presented by us; only two (12.5%) were created through a germinal mosaic (Table 1). Early-onset epilepsy is the most important repeatable feature in all the presented cases (100%). Subject 1 showed epileptic spasms/convulsions [14]. Most boys, as with ours, present tonic, myoclonic, and tonic–clonic epileptic seizures [13]. At the initial stage of the disease, the EEG examination did not differ from the normal one for our boy or for subject 8 [15]. Interictal EEG varies from normal to focal delta slowing in the posterior regions (subjects 4.13) [8,16] to intermittent generalized slowing (subject 10) [8] to focal rapid rhythmic activity (subject 9) [13]. Hypsarrhythmia was presented by subjects 1, 5, 7, 9, and 11 [8,13,14,17]. Slow waves and infantile spasms were noted in subjects 2 and 14 [8,18]. EEG indicated multifocal paroxysmal activity in subject 6 [19] and continuous bihemispheric epileptiform discharges in subject 12 [8]. Brain MRI in our patient showed areas of incomplete myelination, while in six boys (37.5%) this examination was completely normal [13]. In subjects 3, 11, 12, and 15, cerebral atrophy (25%) was observed on MRI [8,20]. In addition, in subjects 9 and 10, there was subarachnoid space widening (12.5%) [13]. Most of the boys had poor eye contact (43.8%), severe global developmental delay (62.5%), and hypotonia (81.3%), sometimes with associated spasticity (25%) [13]. In addition to seizures, our patient had a problem with solid food intake. Subjects 9, 13, and 16 also struggled with dysphagia (18.8%) [8,13]. Regarding additional ailments, for example, subjects 1, 5, and 12 had gastroesophageal reflux (18.8%); subjects 1, 5, and 14 had constipation (18.8%); and subjects 1, 13, and 12 had aspiration pneumonia (18.8%) [8,14]. Along the course of the disease, almost all subjects developed intractable epileptic encephalopathy. The therapy of boys is based on many antiseizure medications, which, as in subject 1 and in our boy, are additionally supported by steroid therapy (12.5%). Of the boys, 93.8% were resistant to antiseizure medications. Only one patient showed a partial response. Attempts were made to stabilize seizures by introducing a ketogenic diet in the boys. Most showed resistance (43.8%) or, like our boy, intolerance. Subject 8 partially responded to the ketogenic diet (6.3%), and subject 13 showed a good response (6.3%) [13]. Only our boy had a vagal nerve stimulator implanted, which was followed by a reduction in the frequency of seizures.

From Table 1, some conclusions can be drawn. In general, the clinical pictures of most of the boys whose cases are collected in the table, including that of our boy, are severe and not well-prognosed. We did not notice a relationship between a given mutation type and the severity of the clinical condition. The clinical picture regarding eye contact, movement disorder, tones, reflexes, and motor skills is similarly severe in all of them, while coexisting symptoms in other organs may be different between patients. Most showed changes in EEGs, and the most common abnormality was hypsarrhythmia. It is worth noting that at the onset of symptoms, EEG changes may not be present [10]. Changes in MRI imaging were present only in some of the boys and were nonspecific, which supports the opinion that MRI neuroimaging studies may not be helpful in diagnosis [7,9]

The treatment of CDKL5 deficiency disorder (CDD) uses both pharmacological treatments and non-pharmacological treatments, such as vagal nerve stimulator implantation. The main aim of treatment is to relieve symptoms and improve the patient’s quality of life. Scientific data on the treatment of patients with CDD are relatively scarce and are mainly limited to the treatment of epilepsy. Most patients have a short-term response to treatment [9]. 

The most commonly used medications include the broad-spectrum antiepileptic medicaments clobazam, valproate, topiramate, levetiracetam, and vigabatrin [7]. The patient response rate to at least one antiepileptic medicament, defined as a 50% reduction in seizures, was 69% at three months, 45% at six months, and dropped to 24% at 12 months [4]. The majority of boys with CDKL 5 mutations do not respond to antiepileptic treatment (Table 1). One boy had a partial response to the antiepileptic medicines phenobarbital, carbamazepine, and vigabatrin, but he never became seizure-free [8]. The boy described by us was also treated with a number of antiseizure medications, such as zonisamide, levetiracetam, valproate, phenytoin, topiramate, vigabatrin, lacosamide from the barbiturate group, phenobarbital, and the benzodiazepine clobazam, but again no long-term clinical improvement was achieved. The boy also received steroid therapy with tetracosactide acetate and methylprednisolone, which had to be stopped due to the appearance of increased dystonic movements and problems with food intake.

The information collected in Table 1 on the treatment of individual cases confirms that CDD is a difficult disease to treat. Practically every boy showed resistance to antiseizure medications, regardless of the type of mutation. Most of them required modifications to the treatments. The use of a ketogenic diet also did not have a positive effect in most cases, but a beneficial effect was noted in two boys. Of the six boys, including ours, in whom steroid therapy was used, only two saw a beneficial effect. This confirms the fact that it is necessary to approach each case individually, because it is impossible to predict how the patient will react to a given treatment. Of all the boys, only ours had a VNS implanted, which may suggest that this is not a common practice in the prevention of epileptic seizures in this disease.

The National Library of Medicine reports that only four boys have been registered with a very similar mutation to our boy. Only one case has been described, so we present another [21]. Table 1 presents the case of the boy described in the article by Mirza, G.M. et al. with the pathogenic variant c.513C > A (p.Tyr171X) [8]. This is the same molecular consequence that occurs in our boy c.513C > G (p.Tyr171X). Both arose as a result of a nonsense mutation, de novo. The onset of epileptic seizures in our boy was noticed at 5 weeks of age and in subject 11 at 5.5 weeks. They both presented epileptic spasms. Additionally, our boy had myoclonic, focal motor seizures, and the boy described by Mirza, G.M. et al. had tonic, tonic–clonic, generalized seizures. The EEG of our boy showed generalized paroxysmal discharges but without the typical bioelectrical silence episodes with normal sleep characteristics. In contrast, the EEG of subject 11 showed hypsarrhythmia. In the MRI scan of our boy, areas of incomplete myelination of the triangles of the lateral ventricles and around the occipital horns were noticeable, and in subject 11 cerebral and cerebellar atrophy with increased extra-axial spaces, widened sulci, ventriculomegaly, and white matter thinning were observed. Both had cortical visual impairment [8]. Additionally, our boy had a problem with maintaining eye contact and suffered from movement disorders. He had hypotonia and contractures in the joints, and the boy described by Mirza, G.M. et al. had limb spasticity axial hypotonia. They both presented a severe global developmental delay. Other significant medical problems include feeding difficulties, with severe gastroesophageal reflux, and secondary respiratory insufficiency, requiring gastrostomy (G-tube) placement in subject 11 [8]. Our boy had a problem only with solid food. Both boys suffered from incurable epilepsy resistant to ASMs. Additionally, subject 11 was resistant to ACTH and the ketogenic diet [8]. In our boy, the ketogenic diet was discontinued because of his reluctance to drink. Steroid therapy was discontinued due to the development of severe dyskinesias and dystonia. Only our boy has a vagal nerve stimulator implanted. According to the literature, at the time of research, subject 11 was 6 years old and most likely he is still alive. Our boy is 2.5 years old, and his condition seems to be more severe than that of the boy described by Mirza, G.M. et al. [8]. Despite the treatment, our boy’s prognosis is very poor. There is no certainty that he will live to the same age as subject 11.

One of the treatment options available is the ketogenic diet. The International CDKL5 Disorders Database collected 204 patients, of whom more than half (*n* = 104) followed a ketogenic diet. The mean time on the diet was 17 months. Of the 69 caregivers who reported changes in seizure activity, 61 (88%) reported positive benefits from the treatment. However, only one-third of the patients ultimately did not quit the ketogenic diet treatment. Patients usually cited lack of efficacy as the reason for quitting [22]. Of the patients collected, two boys are still on the ketogenic diet, but their seizures are not controlled and there is still no good response to treatment [8,16]. One patient had a partial response to treatment with the ketogenic diet, whereas only one patient had significant improvement after treatment [8,15]. The boy described by us was put on a ketogenic diet in February 2022. Unfortunately, it was not well tolerated and was discontinued 5 days after its introduction due to the patient’s general weakness, feeding difficulties, intensification of extrapyramidal dysfunction, and the parents’ refusal to continue the diet.

A less commonly used, nonpharmacological treatment is the implantation of a vagal nerve stimulator (VNS). Only 38 (17%) of 224 individuals in the CDKL5 International Disorders Database had a VNS implanted between the ages of 4 and 9 years [23]. Australian researchers enrolled 38 patients with neurostimulation, whose mean age was 4.9 years, to evaluate the therapy. Improvements in seizure control (in terms of reduced seizure frequency, intensity, and duration) were achieved in more than two-thirds of patients (25/36.69%). Behavioral changes in the form of improved mode were noted in nine patients. Adverse effects occurred in 13% of patients, namely, decreased mode, sleep quality, and decreased food intake [23]. Pediatric trials typically qualify a small number of patients due to the fact that VNS is used when treatment of ASMs does not work in patients. Current data suggest that the earlier the implantation of a VNS in a patient with drug-resistant epilepsy, the better the response to treatment. Pediatric populations have higher overall success rates for VNS therapy than adult populations [24]. This may be related to the greater neuroplasticity of children’s brains than adults. Pediatric patients have benefited from treatment in terms of reduced frequency and severity of epileptic seizures [25]. In 2017, the FDA approved vagal nerve stimulators (VNSs) to be implanted in younger children as well [26]. The presented case had a VNS implanted at the age of 2 years. Initially, the seizure frequency was reduced by half, but after 5 months the seizures became more frequent again. The patient’s caregivers reported an improvement in the boy’s quality of life, but the epileptic seizures have still not been controlled. Implantation of a VNS seems to be a safe and effective form of therapy, with the added benefit of behavioral changes, but it still does not result in complete seizure reduction.

CDD is a condition that causes significant delay in a child’s psychophysical development, so a multidisciplinary approach to care is essential. Unpublished data from the International CDKL5 Disorder Database suggest that caregivers believe that children benefit from physiotherapy and occupational therapies [9]. Patients often struggle with cerebral visual impairment, sleep problems, and gastrointestinal symptoms, which significantly diminish quality of life for both patients and their caregivers [27,28]. That is why participation in psycho-educational classes and therapies is so important. The described patient also receives rehabilitation in the framework of early developmental support and is under the care of many specialists.

Current treatments for the neurological features of CDD are symptom-based and empirical, rather than CDD-specific, although clinical trials for CDD are emerging. Disease-specific and disease-modifying treatments are under development. One recent clinical trial involved ganaxolone, which was approved based on data from the Phase 3 Marigold trial (NCT03572933), a double-blind, placebo-controlled study [29]. The US Food and Drug Administration (FDA) in March 2022 approved ganaxolone for the treatment of seizures associated with CDD in patients aged 2 years and older. This is the first treatment for CDD-related seizures and the first treatment specifically for CDD [30]. Other treatments are in clinical trials. Recent studies have shown reductions in seizures with soticlestat and fenfluramine. Ataluren has not shown efficacy in treating CDD [9,31]. It is worth mentioning that various attempts are being made to develop disease-modifying therapies that address the neurobiology and developmental abnormalities of CDKL5 disorders. These include gene replacement therapy in rodents, using adeno-associated virus. In a recent study published in *Brain*, a team from Imperial developed and tested a therapy that aims to introduce functional *CDKL5* into brain cells lacking a functioning copy of the gene, using a novel serotype of AAV (adeno-associated virus). The therapy has only been tested on mice and human cells, and beneficial results have been obtained. However, the study is still at an early stage, and further work is needed to refine the gene therapy before it can move beyond animal studies. However, the hope remains that it represents a potential pathway for future treatment of this rare disease [32].

## 4. Conclusions

CDKL5 deficiency is a disorder with a heterogeneous phenotype, with a poor prognosis and a more severe clinical course in males. It requires an individualized approach to treatment that often requires modification of antiepileptic therapy as the disease progresses. Failure to respond to antiepileptic medications often results in the need for other methods of treatment, such as the ketogenic diet, vagal nerve stimulation (VNS), or steroid therapy. Currently, no specific therapies are available for people with CDD (apart from ganoxolone, which is not registered in Poland), so medical management is symptomatic and supportive. A multidisciplinary team approach is most effective. Emphasis should be placed on early therapeutic interventions, such as physiotherapy, occupational therapy, and supportive speech and communication therapy. There are few reported cases of vagal nerve stimulation (VNS) treatment in young children, the reason being that the FDA approved this therapy relatively recently. An early-implanted vagal nerve stimulator (VNS) seems to be an opportunity for patients with drug-resistant epilepsy, at least in alleviating the symptoms of the disease and improving quality of life. However, more research is still needed on this subject, with studies including larger groups of described patients, especially patients with VNSs implanted before the age of 5. Considering that genotype–phenotype correlations are still limited for CDD, our data for the affected boy provide additional insights and a better understanding of the variability in the disorder and the possibilities of response to antiseizure medications, the ketogenic diet, steroid therapy, and the use of VNS.

## Figures and Tables

**Table 1:** Molecular characteristics and clinical phenotypes, EEG patterns, and neuroimaging for fifteen reported variants in male subjects with CDD and the presented patient.

Subject	*CDKL5* Variant	Type of Variant	Inheritance	Seizure Onset (Age)	Type of Seizure	EEG Features
1 Weaving, L. S. et al., 2004 [14]	c.183del T p.Met63CysfsX13	Deletion	Parental mosaicism (mother)	4 weeks	Epileptic spasms	Hypsarrhythmia
2 Castrén, M. et al., 2011 [18]	del. 0.3 Mb	Deletion	De novo	5 weeks	Generalized tonic, epileptic spasms, myoclonic	Slow waves and multifocal spasm, diffuse slow waves; multifocal spikes; EEG showed multifocal spikes during sleep
3 Liang, J. S. et al., 2011 [20]	5′UTR region (Xp22.13—no band for exon 1B, thus confirming nullisomy of this region)	Deletion	De novo	1 month	Epileptic spasms	Not available
4 Bartnik, M. et al., 2011 [16]	Xp22.13 del.105 kb	Deletion	De novo	6 weeks	Secondary generalized seizures	Interictal: abnormal slowing of occipital dominant rhythm, multifocal sharp waves, left frontal and central spikes and waves
Subject	*CDKL5* variant	Type of Variant	Inheritance	Seizure Onset (age)	Type of Seizure	EEG Features
5 Mirzaa, G.M. et al., 2013 [8]	c.578A > G p.Asp193Gly	Missense	Germline mosaicism	4 weeks	Tonic, atonic, myoclonic	Hypsarrhythmia; multifocal polyspikes, discontinuous background and periodic burst suppression
6 Mei, D. et al., 2014 [19]	Deletion exon 1	Promoter full deletion (in frame-deletion)	De novo full hemizygous deletion	1 months	Tonic seizures, epileptic spasms	Multifocal paroxysmal activity
7 Arafat, A. et al., 2017 [17]	c.278dupA p.Asn95LysfsX16	Frameshift	De novo	1 month	Focal epileptic spasms	Hypsarrhythmia
8 Neupauerova, J. et al., 2017 [15]	c.2578C > T p.Gln860X	Missense	De novo	1 month	Hypermotor seizures, epileptic spasms, myoclonic, tonic seizures	Normal; then right-sided fronto-temporal spikes; hypsarrhytmia
9 Siri, B. et al., 2021 [13]	c.601_603delCTT p.Leu201del	In-frame deletion	De novo	1 month	Epileptic spasms, focal to bilateral tonic–clonic, focal motor, tonic seizures	Slowing of background activity, unilateral and bilateral focal low-voltage rapid rhythmic activity, followed by discharge of rhythmic spikes and waves or polyspikes; hypsarrhytmia
Subject	*CDKL5* Variant	Type of Variant	Inheritance	Seizure Onset (age)	Type of Seizure	EEG Features
10 Siri, B. et al., 2021 [13]	c.825þ1G > T	Splice site (within intron 10)	De novo	25 days	Focal impairned awareness, epileptic spasms and tonic, myoclonic, hypermotor seizures	Slowing of background activity, waves and spikes and waves on left parieto- temporal regions and diffuse spikes and waves, recruiting diffuse discharges on left fronto-parietal cortex; multifocal spikes on parietal region, atypical hypsarrythmia
11 Mirzaa, G.M. et al., 2013 [8]	c.513C > A p.Tyr171X (exon 8)	Nonsense	De novo	5.5 weeks	Tonic, tonic–clonic, generalized seizures, epileptic spasms	Hypsarrhythmia
12 Mirzaa, G.M. et al., 2013 [8]	c.175C > T p.Arg59X (exon 5)	Nonsense	De novo	6 weeks	Epileptic spasms, myoclonic, tonic seizures	Continuous bihemispheric, epileptiform discharges
13 Mirzaa, G.M. et al., 2013 [8]	c.2593C > T p.Gln865X (exon 13)	Nonsense	De novo	1.5 months	Epileptic spasms, focal, multifocal seizures	Multifocal, epileptiform discharges; recorded generalized seizures
14 Mirzaa, G.M. et al., 2013 [8]	Deletion exon 3	Deletion	De novo	2 weeks	Epileptic spasms, tonic, tonic–clonic, myoclonic seizures	Slow R sharp waves; disorganized, slow, sharp waves, multifocal discharges
15 Mirzaa, G.M. et al., 2013 [8]	c.62A > G p.Glu21Gly (exon 2)	Missense	De novo	5.5 weeks	Epileptic spasms, tonic seizures	Localization related epilepsy, likely arising from the right frontocentral area
16 The present study	c.513C > G p.Tyr171X	Nonsense	De novo	5 weeks	Epileptic seizures, myoclonic, focal motor seizures	The first EEG showed generalized paroxysmal discharges, without the typical bioelectrical silence episodes with normal sleep characteristics. The second EEG showed continuous paroxysmal changes. During video EEG recording, limb tearing and eye rotations were observed
Subject	MRI	Eye Contact, Movement Disorders	Tone/Reflexes	Motor Skills	Comorbidities	Treatment
1 Weaving, L. S. et al., 2004 [14]	Not available	Poor eye contact, movement disorders: severe global developmental delay	Spastic quadriparesis	None	Gastroesophageal reflux, constipation, pneumonia, kyphoscoliosis, hyperventilation	Seizures very difficult to control, despite numerous ASMs (the names of the medicines were not included in the article), trial of adrenocorticotropic hormone, and ketogenic diet
2 Castrén, M. et al., 2011 [18]	Normal	Poor eye contact, movement disorders: severe global developmental delay	Hypotonia	None	Triangular face, facial dysmorphia, ligamentous laxity	Seizures very difficult to control, despite numerous ASMs (rufinamide, oxcarbazepine, clonazepam and levetiracetam), ketogenic diet resistance
Subject	MRI	Eye Contact, Movement Disorders	Tone/Reflexes	Motor Skills	Comorbidities	Treatment
3 Liang, J. S. et al., 2011 [20]	Cerebral atrophy	Severe global developmental delay	Normal	None	Not available	ASM resistance (the names of the medicines were not included in the article)
4 Bartnik, M. et al., 2011 [16]	Normal	Severe global developmental delay	Hypotonia, hyporeflexia	None	No speech	ASM resistance (phenobarbital, levetiracetam, lamotrigine, folinic acid, and pyridoxine sulfate), patient on a ketogenic diet (no information about its effectiveness)
5 Mirzaa, G.M. et al., 2013 [8]	Normal	Poor eye contact, movement disorders: severe global developmental delay	Axial hypotonia Limb spasticity	None	Gastroesophageal reflux, constipation, scoliosis, facial dysmorphia, optic atrophy	Partial response to phenobarbital, carbamazepine, and vigabatrin, but the child was never seizure-free
6 Mei, D. et al., 2014 [19]	Normal	Not available	Quadriparesis with hypotonia	Severe developmental delay	Not available	ASM resistance (the names of the medicines were not included in the article)
7 Arafat, A. et al., 2017 [17]	Ventriculomegaly	Not available	Not available	Severe global developmental delay	Not available	ASMs (oxcarbazepine, carbamazepine, levetiracetam, phenobarbital, topiramate, sodium valproate) and ketogenic diet resistance
Subject	MRI	Eye Contact, Movement Disorders	Tone/Reflexes	Motor Skills	Comorbidities	Treatment
8 Neupauerova, J. et al., 2017 [15]	Mild frontal atrophy	Poor eye contact, no movement disorder	Hypotonia	Severe global developmental delay	Tetralogy of Fallot, sleep problems	ASM (valproic acid, topiramate, phenobarbital, vigabatrin, levetiracetam, phenytoin, clobazam, and their combinations) resistance, partial response to ketogenic diet, adrenocorticotropic hormone partially effective
9 Siri, B. et al., 2021 [13]	Subarachnoid space widening	Poor eye contact, movement disorder	Hypotonia	Severe global developmental delay, motor skills: none	Liquid dysphagia, laryngeal stridor, sleep problems, horizontal nystagmus	ASM (the names of the medicines were not included in the article) and ketogenic diet resistance
10 Siri, B. et al., 2021 [13]	Widening of subarachnoid space and thin corpus callosum, periventricular frontal delayed myelination	Poor eye contact, no movement disorder	Hypotonia	Severe global developmental delay, motor skills: none	Not available	ASM (the names of the medicines were not included in the article) and ketogenic diet resistance
11 Mirzaa, G.M. et al., 2013 [8]	Cerebral and cerebellar atrophy (postnatal microcephaly)	Cortical visual impairment, without movement disorder	Mixed (limb spasticity axial hypotonia)	Severe global developmental delay	Sleep apnea, facial dysmorphism, hypoplastic scrotum, G-tube	Difficult to treat, ketogenic diet and ASM resistance (the names of the medicines were not included in the article), ACTH resistance
Subject	MRI	Eye Contact, Movement Disorders	Tone/Reflexes	Motor Skills	Comorbidities	Treatment
12 Mirzaa, G.M. et al., 2013 [8]	Cerebral and cerebellar atrophy	Cortical visual impairment, with movement disorder	Mixed (limb spasticity, axial hypotonia)	Severe global developmental delay	Gastroesophageal reflux, aspiration pneumonia, growth failure	Difficult to treat, ketogenic diet and ASM resistance (refractory to 17 antiepileptics—the names of the medicaments were not included in the article), responded briefly to treatment with ACTH
13 Mirzaa, G.M. et al., 2013 [8]	Without cerebral atrophy	Cortical visual impairment, with movement disorder	Hypotonia	Severe global developmental delay	Dysphagia, aspiration pneumonia	Difficult to treat, ASM resistance (clorazepate, vigabatrin, levetiracetam, lamotrigine, oxcarbazepine, topiramate), the most effective therapy was topiramate, ACTH resistance, ketogenic diet with good response
14 Mirzaa, G.M. et al., 2013 [8]	Without cerebral atrophy	Cortical visual impairment, with movement disorder	Severe hypotonia	Severe global developmental delay	Neurogenic bladder, G-tube, constipation	Difficult to treat, ASM resistance (the names of the medicines were not included in the article)
15 Mirzaa, G.M. et al., 2013 [8]	Cerebral atrophy	Cortical visual impairment, without movement disorder	Severe hypotonia	Severe global developmental delay	G-tube, osteopenia, respiratory insufficiency	Difficult to treat, ASM resistance (the names of the medicines were not included in the article)
Subject	MRI	Eye Contact, Movement Disorders	Tone/Reflexes	Motor Skills	Comorbidities	Treatment
16 The present study	Areas of incomplete myelination (in the area of the triangles of the lateral ventricles and around the occipital horns)	Cortical visual impairment, poor eye contact, with movement disorder	Hypotonia, joint contractures	Severe global developmental delay	Dysphagia (only liquid food)	Difficult to treat, ASM resistance (valproic acid, topiramate, vigabatrin, phenobarbital, levetiracetam, phenytoin, clobazam, clonazepam, nitrazepam, lamotrigine, carbamazepine, and zonisamide), steroid therapy (adrenocorticotropic hormone, methylprednisolone—break), vagal nerve stimulation, ketogenic diet intolerance (due to reluctance to drink)

## Data Availability

All data and material analyzed in this study are included in the published article.
